# Cardiovascular outcomes after curative prostate cancer treatment: A population-based cohort study

**DOI:** 10.3389/fonc.2023.1121872

**Published:** 2023-03-31

**Authors:** Camilla Kjellstadli, Rachel B. Forster, Tor Å. Myklebust, Tone Bjørge, Kaare H. Bønaa, Svein I. Helle, Rune Kvåle

**Affiliations:** ^1^ Department of Health Registry Research and Development, Norwegian Institute of Public Health, Bergen, Norway; ^2^ Department of Oncology and Medical Physics, Haukeland University Hospital, Bergen, Norway; ^3^ Department of Registration, Cancer Registry of Norway, Oslo, Norway; ^4^ Department of Research and Innovation, Møre and Romsdal Hospital Trust, Ålesund, Norway; ^5^ Department of Global Public Health and Primary Care, University of Bergen, Bergen, Norway; ^6^ Section for Cervical Cancer Screening, Cancer Registry of Norway, Oslo, Norway; ^7^ Department of Cardiology, St Olav’s Hospital, Trondheim University Hospital, Trondheim, Norway; ^8^ Department of Circulation and Medical Imaging, Norwegian University of Science and Technology, Trondheim, Norway

**Keywords:** prostate cancer, radical prostatectomy, definitive radiotherapy, cardiovascular disease, curative treatment, epidemiology, registry

## Abstract

**Objective:**

To investigate differences in cardiovascular disease (CVD) morbidity and mortality after radical prostatectomy or definitive radiotherapy with or without androgen deprivation therapy (ADT).

**Materials and methods:**

We used population-based data from the Cancer Registry of Norway, the Norwegian Patient Registry and the Norwegian Cause of Death Registry including 19 289 men ≤80 years diagnosed with non-metastatic prostate cancer during 2010-2019. Patients were treated with radical prostatectomy or definitive radiotherapy. We used competing risk models to compare morbidity from overall CVD, acute myocardial infarction (AMI), cerebral infarction, thromboembolism, and CVD-specific mortality for the overall cohort and stratified by prognostic risk groups.

**Results:**

After a median follow-up time of 5.4 years (IQR 4.6 years), there were no differences in adjusted rates of AMI, cerebral infarction, and CVD-specific death between radical prostatectomy and definitive radiotherapy in any of the prognostic risk groups. Rates of overall CVD (0.82; 95% CI 0.76-0.89) and thromboembolism (0.30; 95% CI 0.20-0.44) were lower for definitive radiotherapy than radical prostatectomy during the first year of follow-up. After this overall CVD rates (1.19; 95% CI 1.11-1.28) were consistently higher across all risk groups in patients treated with definitive radiotherapy, but there were no differences regarding thromboembolism.

**Conclusions:**

During the first years after treatment, no differences were found in rates of AMI, cerebral infarction, and CVD-specific death between radiotherapy and radical prostatectomy in any of the prognostic risk groups. This suggests that ADT use in combination with radiotherapy may not increase the risks of these outcomes in a curative setting. The increased overall CVD rate for definitive radiotherapy after the first year indicates a possible relationship between definitive radiotherapy and other CVDs than AMI and cerebral infarction.

## 1 Introduction

Prostate cancer patients diagnosed today have comparable survival to the general population (1). Many of them have cardiovascular comorbidity at time of diagnosis or may develop cardiovascular disease (CVD) after being diagnosed with prostate cancer (2–4). Cancer itself may increase the risk of CVD, and the diseases share many risk factors (5, 6). Additionally, both non-cardiac surgery and radiotherapy may increase the risk of CVD through complex mechanisms, for example by inducing systemic inflammation and immune modulation (7–9).

Radical prostatectomy and definitive radiotherapy with or without androgen deprivation therapy (ADT) are the main curative treatment options for prostate cancer (10). Previous observational studies have found an association between ADT and increased risk of CVD and death, which is not fully corroborated in secondary analyses from randomized controlled trials (11–15). ADT is provided in combination with radiotherapy in prostate cancer patients with intermediate and high risk of disease relapse, while patients with low risk of relapse treated with radiotherapy and those treated with radical prostatectomy do not receive ADT (10). Adherence to guidelines for curative treatment of prostate cancer is generally high in Norway (16, 17), providing an opportunity to use prognostic risk groups to compare influence of ADT use in non-metastatic prostate cancer when detailed prescription data is not fully available.

Few studies have compared CVD morbidity and mortality between patients treated with radical prostatectomy or definitive radiotherapy in non-metastatic prostate cancer exclusively. Wallis et al. found that treatment with radiotherapy and ADT each independently increased risks of ischemic cardiac disease and CVD-specific mortality in non-metastatic prostate cancer patients ≥65 years compared to radical prostatectomy (18, 19). Guo et al. also found increased CVD-specific mortality in radiotherapy patients (20). Neither analyzed dosage or duration of radiotherapy nor included patients from the last decade.

More recently, we have seen increased use of curative treatment for high risk prostate cancer and older patients, and more use of active surveillance in lower risk disease (21). Simultaneously, CVD risk factors, -morbidity, and -mortality have decreased in the general population (22, 23). Additionally, improvements in prostate cancer treatment have been introduced, such as intensity-modulated-, volumetric arc- and image-guided radiation therapy as well as dose escalation, moderate hypofractionation and robot-assisted surgery (10). These changes warrant an updated comparison of CVD after curative treatment for non-metastatic prostate cancer.

We hypothesized that there are no differences in CVD morbidity or mortality following radical prostatectomy or definitive radiotherapy in non-metastatic prostate cancer after adjusting for known confounders, such as age and comorbidity. The aims of the current study were i) to compare CVD morbidity after radical prostatectomy with CVD morbidity after definitive radiotherapy stratified by prostate cancer prognostic risk group and ii) to investigate if there are differences in cardiovascular mortality between patients treated with radical prostatectomy or definitive radiotherapy with or without ADT.

## 2 Materials and methods

**Figure 1:**
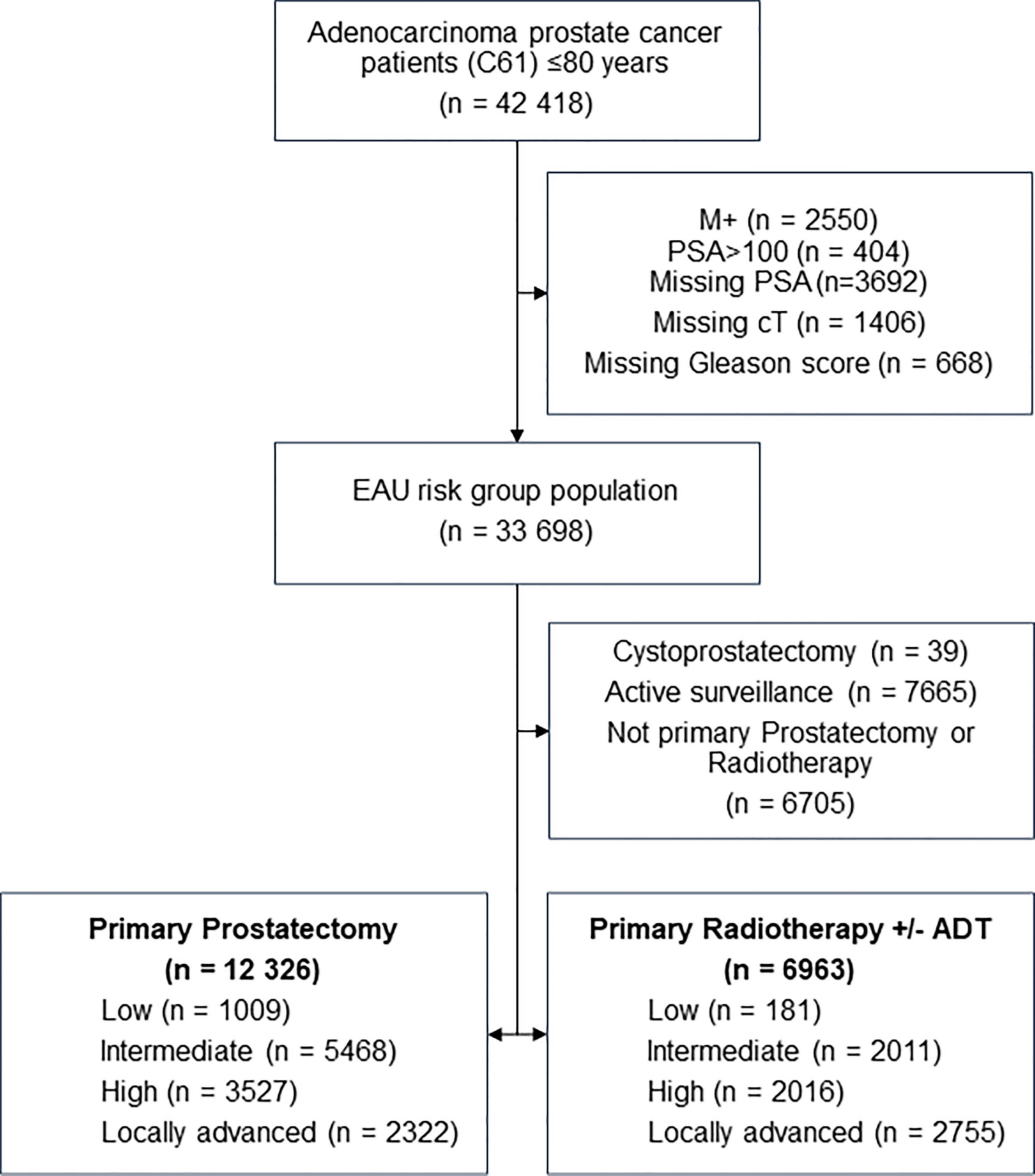
Study population flow chart.

All men ≤80 years diagnosed with prostate adenocarcinomas during 2010-2019 were identified from the Cancer Registry of Norway (CRN) and linked with data from the Norwegian Patient Registry (NPR) and the Norwegian Cause of Death Registry (NCoDR). We excluded patients with distant metastases, PSA >100, or missing information on PSA, clinical T-category (cT) or Gleason score. Patients diagnosed after cystoprostatectomy or fulfilling criteria for active surveillance (≤cT2a, M0, Gleason score ≤7a and PSA <10 ng/ml), not treated with radical prostatectomy within six months or definitive radiotherapy within 12 months after diagnosis were also excluded, due to missing information about possible ADT treatment in this group. 19 289 patients with complete information for risk grouping (European Association of Urology (EAU) guidelines) who underwent definitive radiotherapy or radical prostatectomy were included (**Figure 1**) (10).

CRN provided information about the patients’ age at diagnosis, date of diagnosis, cTNM, Gleason score in biopsy, PSA at diagnosis, date of prostatectomy or start of definitive radiotherapy, target site and radiation dose, WHO performance status and Norwegian health care region (24). Curative treatment was defined as radical prostatectomy within six months after diagnosis or definitive radiotherapy with a target dose of ≥74 Gy in 2 Gy units with treatment start within 14 months after diagnosis.

Patients were divided into four prognostic risk groups according to EAU guidelines, which define low risk prostate cancer as PSA <10 ng/ml and Gleason score <7 and cT1-2a; intermediate risk as PSA 10-20 ng/ml or Gleason score 7 or cT2b; high risk localized as PSA >20 ng/ml or Gleason score >7 or cT2c; and high risk locally advanced as cT3-4 or N1 with any PSA and Gleason score (10).

Norwegian treatment guidelines for prostate cancer generally follow EAU guidelines, recommending curative treatment with radical prostatectomy or definitive radiotherapy (10, 25). ADT is provided in combination with definitive radiotherapy according to prognostic risk group at diagnosis, which we used as a proxy for ADT duration: 1) low risk: no ADT; 2) intermediate risk: six months ADT; 3) high risk localized: 18-24 months ADT and 4) high risk locally advanced: ≥24 months ADT (10, 25). Since actual treatment may deviate from guidelines e.g., due to drug side effects or patient preferences, our use of ADT proxy should be interpreted as an intention-to-treat.

NPR provided information about diagnoses from hospital visits and private specialist outpatient visits based on International Classification of Diseases (ICD)-10 codes. NPR hospital diagnoses were used for morbidity outcomes, which were overall CVD, acute myocardial infarction (AMI), cerebral infarction and thromboembolism (see **Supplemental File**). Previous CVD was based on all available diagnoses in NPR within two years before the prostate cancer diagnosis. The NCoDR provided information about underlying cause and date of death used for CVD-specific mortality.

Charlson comorbidity index was based on hospital diagnoses within two years before the prostate cancer diagnosis, excluding the prostate cancer diagnosis. A higher score indicated more comorbidity and was categorized as 0, 1, 2 or ≥3. WHO performance status was categorized as 0, 1 or ≥2; a higher score indicates poorer functional status (24). Previous cancer included any cancer diagnosis prior to the prostate cancer diagnosis, except non-melanoma skin cancer. Place of treatment was divided into four health care regions within Norway (West, South-East, Central and North).

### 2.1 Statistical analyses

Baseline characteristics were described as median and interquartile range (IQR) for continuous variables and counts and percentages for categorical variables. Differences between treatment groups were tested with Wilcoxon rank sum test for continuous variables and Chi square test for categorical variables. Follow-up time was calculated from time of treatment until death or censoring on December 31, 2020.

Survival time was defined from start of treatment with radical prostatectomy or definitive radiotherapy until patients experienced an event: overall CVD, AMI, cerebral infarction, thromboembolism, death, or censoring on December 31, 2020. We calculated cumulative incidence of overall CVD, AMI, cerebral infarction, thromboembolism, and CVD-specific death by treatment group using the Aalen-Johansen estimator. We estimated Cox proportional hazard models and Fine and Gray competing risk models, accounting for death as a competing risk in morbidity outcomes and deaths from all other causes for CVD-specific mortality. The proportional hazards assumption was examined using Schoenfeld residuals. An interaction term between time and treatment and/or previous CVD was used when the proportional hazards assumption was found to be invalid. Covariates in adjusted analyses were age, cT, Gleason score in biopsy, PSA, previous CVD, Charlson comorbidity index, WHO performance status, previous cancer, health care region and prostate cancer diagnosis year. Analyses were performed for the entire cohort and stratified by EAU risk group.

For sensitivity analysis, we repeated all analyses starting follow-up from 14 months after diagnosis, due to differences in time from diagnosis to treatment. We performed stratified analyses based on previous CVD status to check whether this influenced our results in the overall cohort and in the high risk groups. We assessed whether postoperative radiotherapy (adjuvant or salvage) influenced all outcomes in the entire cohort or was an independent risk factor for CVD. For CVD in the entire cohort, we repeated the analysis starting follow-up one month after treatment. We did not perform a separate subgroup analysis in the low risk group for CVD-specific deaths, due to a low number of deaths.

Results are presented as unadjusted cumulative incidence plots and adjusted cause-specific hazard ratios (aCSHR) with 95% CI from Cox regression models. Subdistribution hazard ratios are presented in **Supplementary Tables**. Two-sided p-values <0.05 were considered statistically significant in all analyses. Analyses were conducted with Stata version 17.0.

## 3 Results

Overall, 12 326 (63.9%) patients underwent radical prostatectomy and 6963 (36.1%) definitive radiotherapy (**Figure 1**). Median follow-up time after treatment was 5.4 years (IQR 4.6, range 0.0-11.0 years), 5.5 years (IQR 4.7) for radical prostatectomy and 4.6 years (IQR 4.6) for definitive radiotherapy. In total, 2333 patients initially treated with radical prostatectomy later received postoperative radiotherapy (adjuvant or salvage) and 25 definitive radiotherapy patients later underwent radical prostatectomy. The number of patients diagnosed were relatively stable over time, but the percentage of definitive radiotherapy patients decreased from 40.1% of all patients in 2010-2011 to 31.5% in 2018-2019 (**Supplementary Figure 1**). As expected, radical prostatectomy patients were younger than definitive radiotherapy patients (median 64 years vs. 71 years), had a higher proportion of lower risk disease, less comorbidity and better functional status (**Table 1**).

**Table 1:** Baseline characteristics^1^.

	Prostatectomy (n=12326)	Radiotherapy (n=6963)
Diagnosis year, n (%)		
2010-2011	2193 (17.8)	1471 (21.1)
2012-2013	2571 (20.9)	1538 (22.1)
2014-2015	2608 (21.2)	1486 (21.3)
2016-2017	2491 (20.2)	1336 (19.2)
2018-2019	2463 (20.0)	1132 (16.3)
**Age y, median (IQR)**	64 (8)	71 (8)
EAU Risk group^2^, n (%)		
Low	1009 (8.2)	181 (2.6)
Intermediate	5468 (44.4)	2011 (28.9)
High, localized	3527 (28.6)	2016 (29.0)
High, locally advanced	2322 (18.8)	2755 (39.6)
cT stage, n (%)		
T1-2a	7163 (58.1)	2887 (41.5)
T2b	1146 (9.3)	569 (8.2)
T2c	1763 (14.3)	793 (11.4)
T3-4	2254 (18.3)	2714 (39.0)
N stage, n (%)		
N0	5902 (47.9)	3548 (51.0)
N1	175 (1.4)	288 (4.1)
NX	6249 (50.7)	3127 (44.9)
Gleason score, n (%)		
≤6	1888 (15.3)	664 (9.5)
7a	4950 (40.2)	2023 (29.1)
7b	2831 (23.0)	1599 (23.0)
8	1787 (14.5)	1498 (21.5)
9-10	870 (7.1)	1179 (16.9)
PSA (ng/ml), n (%)		
0-9.9	8079 (65.5)	2957 (42.5)
10.0-20.0	3247 (26.3)	2450 (35.2)
20.1-100.0	1000 (8.1)	1556 (22.4)
WHO performance status, n (%)		
0	9744 (79.1)	4707 (67.6)
1	782 (6.3)	1293 (18.6)
≥2	106 (0.9)	283 (4.1)
Missing	1694 (13.7)	680 (9.8)
Charlson comorbidity index^3^, n (%)		
0	10472 (85.0)	4871 (70.0)
1	1213 (9.8)	1218 (17.5)
2	502 (4.1)	583 (8.4)
≥3	139 (1.1)	291 (4.2)
Previous other cancer, n (%)		
Yes	689 (5.6)	655 (9.4)
No	11637 (94.4)	6308 (90.6)

^1^Chi square test for categorical variables and Wilcoxon rank sum test for continuous variables were performed to test for differences in distribution. For all differences, p-value was below 0.0001. ^2^Risk groups defined according to the European Association of Urology (EAU) Guidelines.

Before their prostate cancer diagnosis, 29.1% of definitive radiotherapy patients had previous CVD, compared to 15.4% of radical prostatectomy patients (**Table 2**). A higher proportion of definitive radiotherapy patients had previous AMI (1.9% vs 0.8%), cerebral infarction (1.5% vs 0.5%) or thromboembolism (1.0% vs 0.6%).

**Table 2:** Cardiovascular disease before diagnosis of prostate cancer (baseline) and after treatment with prostatectomy or radiation.

	Baseline^1^				Follow-up			
	Total (n=19289)	Prostatectomy (n=12326)	Radiotherapy (n=6963)	p-value^2^	Total (n=19289)	Prostatectomy (n=12326)	Radiotherapy (n=6963)	p-value^2^
**CVD, n (%)**	3918 (20.3)	1895 (15.4)	2023 (29.1)	<0.001	8554 (44.4)	4797 (38.9)	3757 (54.0)	<0.001
**AMI, n (%)**	236 (1.2)	103 (0.8)	133 (1.9)	<0.001	850 (4.4)	456 (3.7)	394 (5.7)	<0.001
**Cerebral infarction, n (%)**	159 (0.8)	58 (0.5)	101 (1.5)	<0.001	643 (3.3)	314 (2.6)	329 (4.7)	<0.001
**Thromboembolism, n (%)**	135 (0.7)	68 (0.6)	67 (1.0)	0.001	578 (3.0)	357 (2.9)	221 (3.2)	0.227

^1^Based on information until 2 years prior to PCa diagnosis. ^2^Pearson chi-square test.

After treatment, a higher proportion of definitive radiotherapy patients had a new CVD event (54.0% vs 38.9%), including AMI (5.7% vs 3.7%) and cerebral infarction (4.7% vs 2.6%). Thromboembolic events were similar in the two treatment groups (3.2% vs 2.9%).

### 3.1 Overall cardiovascular disease

**Figure 2:**
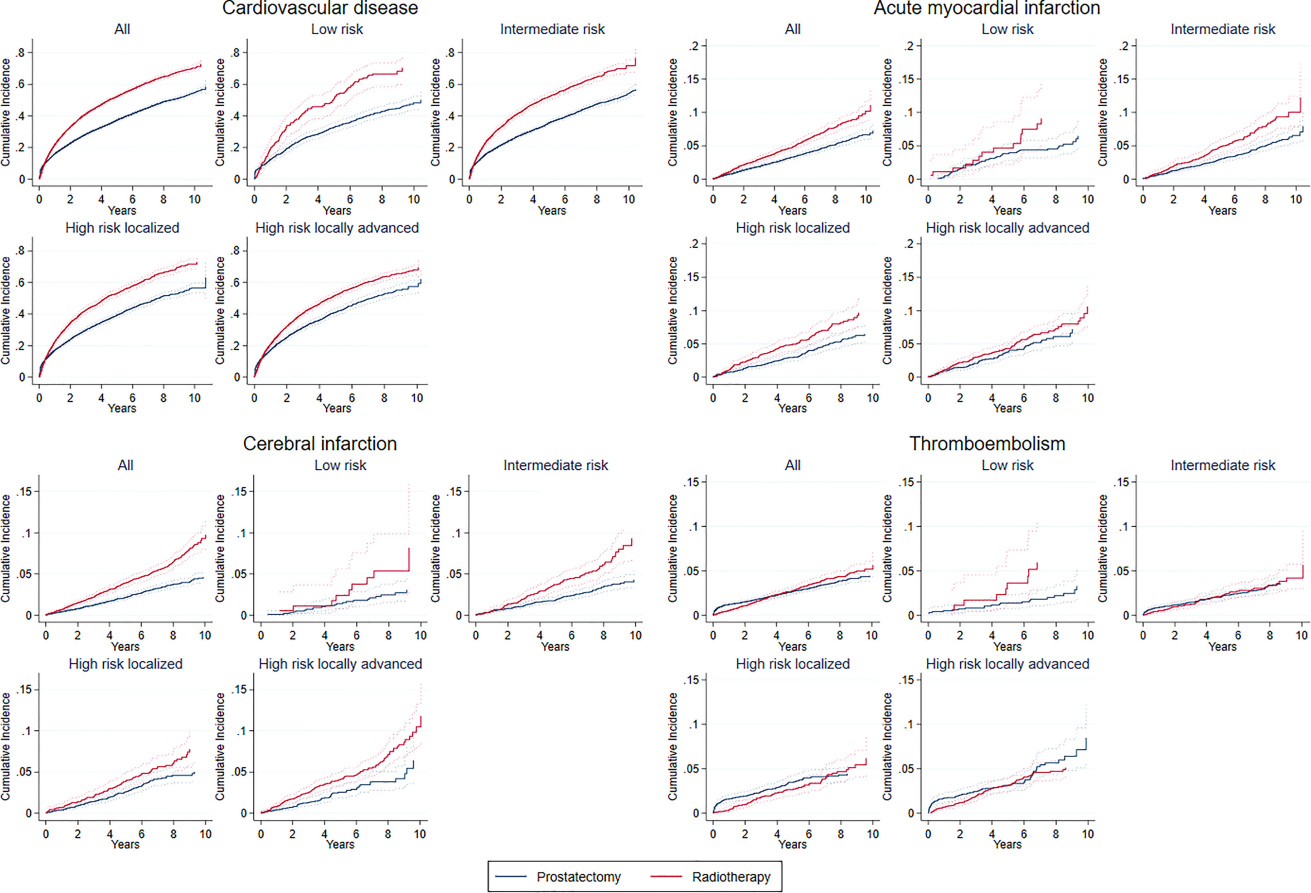
Unadjusted cumulative incidence of overall cardiovascular disease (CVD), acute myocardial infarction (AMI), cerebral infarction and thromboembolism in total cohort and by risk groups. Risk groups defined according to the European Association of Urology (EAU) Guidelines. Years correspond to years since date of radical prostatectomy or start date of definitive radiotherapy.

**Figure 3:**
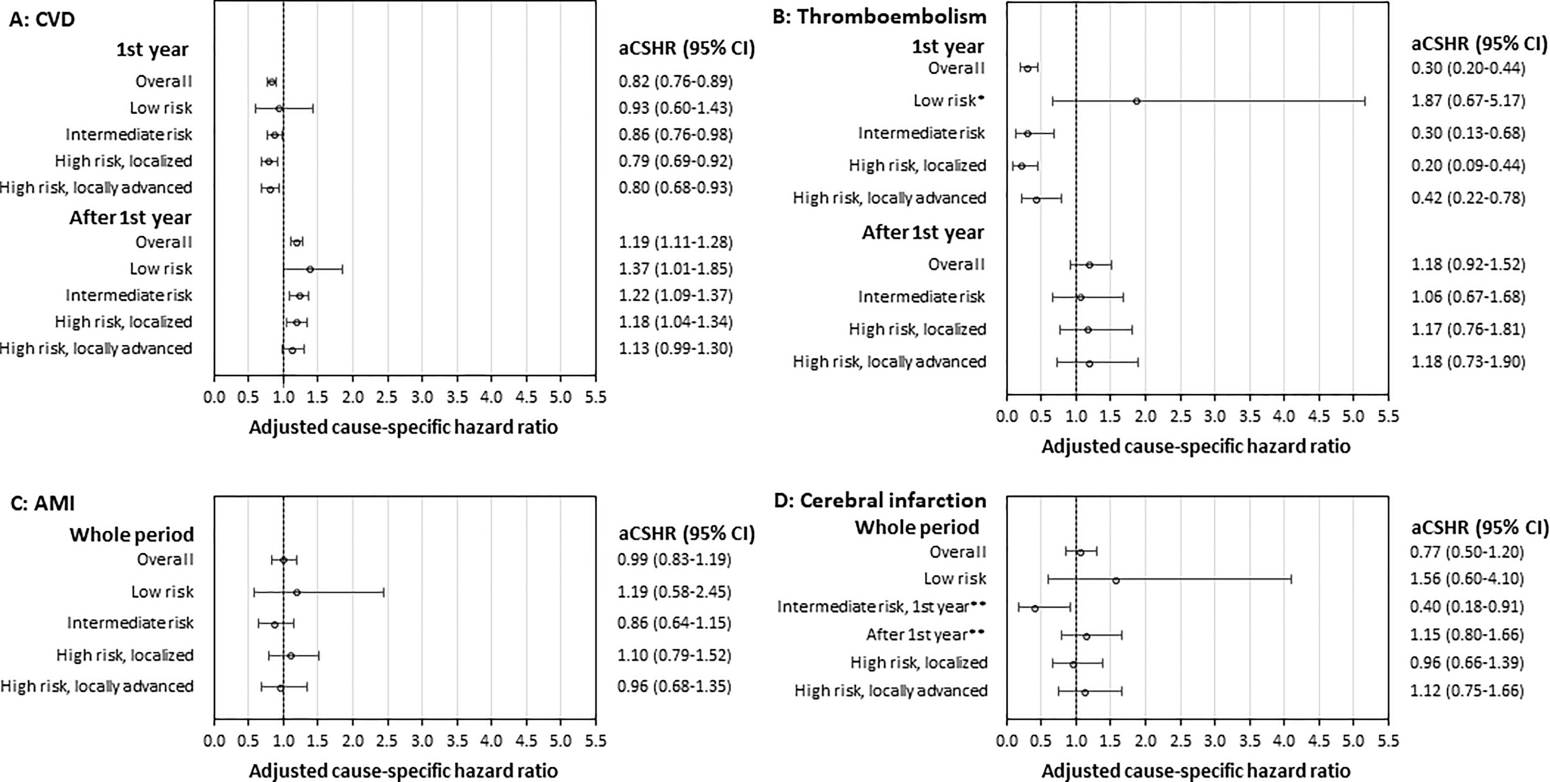
Adjusted cause-specific hazard ratios (aCSHR) for cardiovascular event **(A)** CVD; **(B)** Thromboembolism; **(C)** AMI; and **(D)** Cerebral infarction, comparing treatment radiotherapy to prostatectomy. Adjusted for age, previous cardiovascular disease, cT stage, cN stage, Gleason score, PSA, Charlson comorbidity index, WHO performance status, healthcare region, diagnosis year. Risk groups defined according to the European Association of Urology (EAU) Guidelines.*1^st^ year and after 1^st^ year combined. **Intermediate risk divided into two time periods due to proportional hazards assumption found to be invalid.

Radical prostatectomy patients had a higher unadjusted cumulative incidence of overall CVD immediately after treatment but were surpassed by CVD events in the definitive radiotherapy group within six months of treatment (**Figure 2**). Definitive radiotherapy patients had a lower adjusted rate of CVD events in the first year compared to radical prostatectomy patients in the entire cohort (aCSHR 0.82; 95% CI 0.76-0.89), and similarly in intermediate, high risk localized and, locally advanced risk groups (**Figure 3**). Postponing the start of follow-up one month after treatment start, led to more CVD events for definitive radiotherapy in the entire cohort during the first year in adjusted analyses (**Supplementary Table 1**). After the first year, definitive radiotherapy patients had a higher adjusted rate of CVD events in the entire cohort and across all risk groups. *Post-hoc* analyses indicated a possible relationship with definitive radiotherapy and peripheral arterial disease. Radiotherapy was associated with increased risk of peripheral arterial disease after treatment compared to surgery (HR 1.70, 95% CI 1.39-2.07) with no major differences across risk groups or in stratified analyses based on previous CVD status. Increasing age was associated with a 17% increase in the CVD rate per 5-year increase in age (**Supplementary Table 2**). Previous CVD was associated with a new CVD event (total cohort 1^st^ year aCSHR 3.14; 95% CI 2.90-3.39 and after 1^st^ year aCSHR 1.19; 95% CI 1.11-1.28), following the same pattern in stratified analyses. No clear differences in rates of overall or specific CVDs were found when comparing people with previous CVD to those without previous CVD in the entire cohort, or in the higher risk groups (**Supplementary Table 3**).

### 3.2 Acute myocardial infarction

The unadjusted cumulative incidence of AMI was higher for definitive radiotherapy patients (**Figure 2**). We found no differences in the aCSHR of AMIs comparing radical prostatectomy and definitive radiotherapy in the whole cohort or in stratified analyses (**Figure 3**). Previous CVD was associated with an increased aCSHR of AMI (aCSHR 1.35; 95% CI 1.13-1.61) in the entire cohort (**Supplementary Table 2**).

### 3.3 Cerebral infarction

The unadjusted cumulative incidence of cerebral infarction was higher for definitive radiotherapy patients (**Figure 2**). There were no differences in aCSHR of cerebral infarction in the total cohort or in stratified analyses, except for intermediate disease (**Figure 3**). Intermediate risk definitive radiotherapy patients had a lower rate of cerebral infarction in the first year of follow-up (aCSHR 0.40; 95% CI 0.18-0.91). In the entire cohort, previous CVD was associated with an increased aCSHR of cerebral infarction, with similar findings in stratified analyses (**Supplementary Table 2**).

### 3.4 Thromboembolism

The unadjusted cumulative incidence of thromboembolism increased immediately after radical prostatectomy and remained higher than for definitive radiotherapy for nearly four years (**Figure 2**). Later, definitive radiotherapy patients had a higher unadjusted cumulative incidence of thromboembolism. The aCSHR of thromboembolism was consistently lower for definitive radiotherapy patients in the first year of follow-up, apart from low risk disease (**Figure 3**). Previous CVD was associated with thromboembolism the first year after treatment (**Supplementary Table 2**).

### 3.5 Mortality

**Figure 4:**
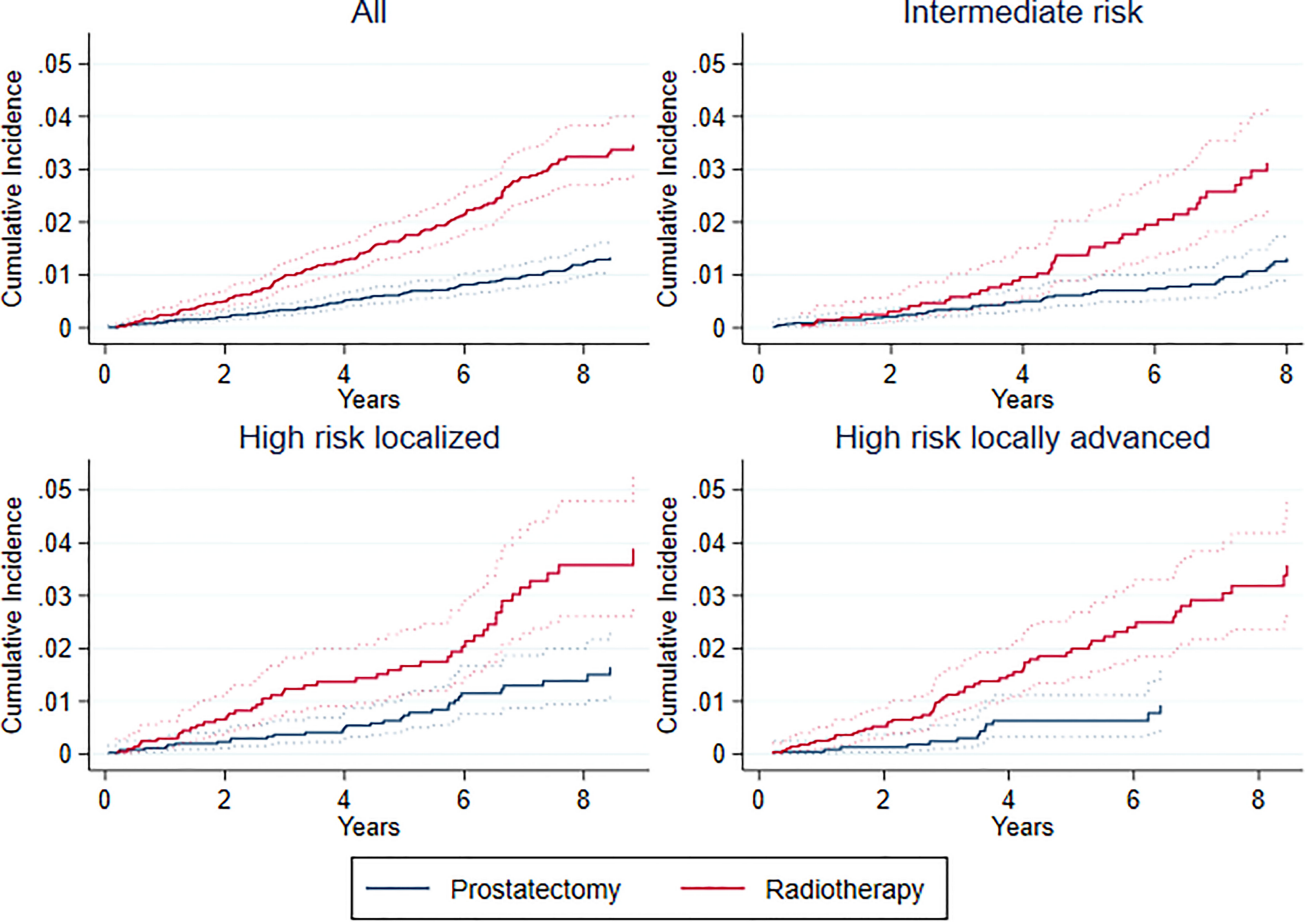
Cumulative incidence of cardiovascular-specific death in total cohort and by risk groups. Risk groups defined according to the European Association of Urology (EAU) Guidelines: 1) intermediate risk: PSA 10-20 ng/ml or Gleason score 7 or cT2b; 2) high risk localized: PSA >20 ng/ml or Gleason score >7 or cT2c; 3) high risk locally advanced: cT3-4 or N1 with any PSA and Gleason score. Years correspond to years since date of radical prostatectomy or start date of definitive radiotherapy.

Overall, 1235 (6.4% of all) patients died during follow-up, of which 241 (19.5% of all deaths) were from CVD and 252 (20.4%) from prostate cancer. Unadjusted cumulative incidence of CVD-specific death was higher for definitive radiotherapy patients (**Figure 4**). There were no statistically significant differences in aCSHR for the entire cohort or in stratified analyses (**Table 3**). Postoperative radiotherapy did not influence our finding of no difference between treatment groups regarding CVD-specific deaths. Previous CVD was associated increased risk (aCSHR (CI): 1.56 (1.13-2.14)) of CVD specific mortality in the total study population (**Supplementary Table 2**).

**Table 3:** Cardiovascular deaths and cause-specific hazard ratios (aCSHR) comparing radiotherapy to prostatectomy (reference). .

Risk group	No of CVD deaths/total no patients (%)		Adjusted CSHR (95% CI)
	Prostatectomy	Radiotherapy	
**Total**	95/12326 (0.8)	146/6963 (2.1)	1.12 (0.79-1.59)
**Low risk**	7/1009 (0.7)	3/181 (1.7)	–
**Intermediate risk**	43/5468 (0.8)	40/2011 (2.0)	1.19 (0.68-2.10)
**High risk, localized**	32/3527 (0.9)	47/2016 (2.3)	0.92 (0.50-1.69)
**High risk, locally advanced**	13/2322 (0.6)	56/2755 (2.0)	1.17 (0.57-2.41)

Adjusted for age, previous cardiovascular disease, cT stage, cN stage, Gleason score, PSA, Charlson comorbidity index, WHO performance status, healthcare region, diagnosis year. Risk groups defined according to the European Association of Urology (EAU) Guidelines.

## 4 Discussion

We found that patients treated with definitive radiotherapy had a higher unadjusted cumulative incidence of AMI, cerebral infarction, and CVD-specific death than patients treated with radical prostatectomy. During the first year, unadjusted cumulative incidence and adjusted rates of CVD and thromboembolism were higher after radical prostatectomy. After the first year, definitive radiotherapy was associated with a higher adjusted rate of overall CVD across all prognostic risk groups, but there were no differences between treatment groups regarding thromboembolism. There were no consistent differences in adjusted rates of AMI or cerebral infarction after definitive radiotherapy or radical prostatectomy, also none in the intermediate or high risk groups, where patients treated with definitive radiotherapy receive ADT. During follow-up, 20% of deaths were from CVD and 20% from prostate cancer and there were no differences in CVD-specific mortality rates between treatment groups.

Earlier studies have mainly focused on ADT and risk of CVDs, with wide variation in outcome measures, ADT types, prostate cancer stages and comparator groups (11, 26, 27). The relationship between ADT and overall CVD, AMI and stroke is still unresolved, although results from previous studies lean towards ADT increasing the risk of these outcomes (11, 26, 27). Our findings partly contradict this for curatively treated patients, where ADT is combined with definitive radiotherapy. We found an increased rate of overall CVD for definitive radiotherapy starting after the first year of follow-up, and no consistent difference by treatment type in the rate of AMI or cerebral infarction. This is also partly in contrast to two previous studies of older, non-metastatic prostate cancer patients that found increased risk of CVD and AMI for definitive radiotherapy compared to radical prostatectomy (18, 19). Systemic inflammation caused by radiotherapy may have contributed to the increased risk of CVD (8, 9) and pelvic radiotherapy may, based on case reports, lead to peripheral arterial disease (8). Our results indicated higher risk of peripheral arterial disease among patients treated with radiotherapy compared to surgery across all risk groups. However, as numbers were relatively small and details regarding radiotherapy techniques (prostate only versus whole pelvic radiotherapy) were lacking, we cannot conclude whether the observed increased risk can be explained by side effects of radiation. We may also have residual confounding if baseline characteristics are insufficiently accounted for.

We found no differences in rates of overall or specific CVDs across risk groups. In addition, there were no clear differences between treatment groups for these outcomes when comparing people with previous CVD to those without previous CVD overall, or in the higher risk groups. These results may indicate that ADT duration up to two years does not increase risk of CVD when treated in a curative setting. However, deviation from guidelines e.g. after clinical judgement of the individual patient may occur. Caution is therefore needed when interpreting the results based on risk groups as proxy for ADT duration. Better CVD prevention in more recent years may also have mitigated a potential relationship between ADT use and later CVD (22, 23).

The risk of thromboembolism increased immediately after radical prostatectomy, and these patients were at higher risk of thromboembolism compared to definitive radiotherapy patients until one year post-treatment. Similar findings for overall CVD disappeared when start of follow-up was postponed one month, indicating thromboembolic complications after radical prostatectomy may be a possible explanatory factor. In accordance with our findings, Van Hemelrijck et al. proposed increased risk of thromboembolism after radical prostatectomy versus definitive radiotherapy (28). Other studies have found increased risk of thromboembolism in prostate cancer patients in general, especially with ADT treatment, and longer duration of ADT (11, 28, 29).

Risk of thromboembolism is higher with open radical prostatectomy compared to robot-assisted and laparoscopic surgery, more extended lymph node dissection and increased patient baseline risk, which is generally reflected in thromboprophylaxis guidelines (25, 30, 31). Adherence to guidelines varies within and between countries (30). In our study, robot-assisted radical prostatectomy increased from roughly half of prostatectomies performed in 2010-2011 to nearly all in 2018-2019. Risk of thromboembolism did not decrease in recent years as would be expected with less invasive treatment. While this emphasizes a need to focus on thromboprophylaxis for people undergoing radical prostatectomy, only 140 of 12 326 (1.1%) prostatectomized patients experienced a thromboembolic event within 90 days of surgery.

Prostate cancer patients have good prognosis, which increases chances of dying from CVD (5). The proportion of people who died from CVD and prostate cancer was similar in our population, both 20% of all deaths. A large US study had comparable findings with 17% prostate cancer-specific deaths and 23% CVD-specific deaths (2), but varied in other studies (32, 33). The patients with previous CVD had around 50% higher risk of CVD-specific mortality. These patients may benefit from more intensive CVD prevention measures.

We found no association between treatment type and CVD-specific mortality overall or across risk groups. The few studies specifically comparing radical prostatectomy to definitive radiotherapy found an increased risk of CVD-specific deaths with definitive radiotherapy but are not directly comparable as the data were older and the study populations were different in terms of age composition and disease characteristics (18–20). Other recent studies found that non-metastatic prostate cancer patients treated with ADT were not at higher risk of dying from CVD compared to non-ADT treatment, while older reviews have mixed results (11, 12, 14, 32–34).

Limitations of our study include lack of specific information about type, timing, or duration of ADT. Further information about factors such as socioeconomic factors, risk-modifying treatment for CVD or complete information about CVD risk factors, which are highly prevalent in the prostate cancer population (35) are also lacking. Traditionally, older patients and those with more comorbidities are treated with definitive radiotherapy, while younger patients with less comorbidity are more often treated with radical prostatectomy. We adjusted for age, comorbidity from hospital and private specialist diagnoses and functional status, which should account for much of this selection mechanism, but we may still have residual confounding. However, we propose that lack of sufficient information would affect the older patients with comorbidities more, which makes a null finding of no difference in outcomes between treatment groups more convincing.

Considering effects of postoperative radiotherapy did not change our conclusions for any of the outcomes, but we could not account for other secondary treatments. Follow-up time differed for the treatment groups because radical prostatectomies are generally performed closer to diagnosis than definitive radiotherapy. We could not specify ADT timing further as it varied during the study period and between Norwegian hospitals. Sensitivity analysis performed by starting follow-up 14 months from diagnosis for everyone did not influence our findings. Generally, results in stratified analyses follow the overall cohort, but were often not statistically significant. This may be explained by lack of power due to small group sizes. This is evident in the low risk group, where only 181 of 1190 patients were treated with definitive radiotherapy. Ideally, we should have a longer follow-up time than a median of five years, especially for CVD-specific mortality.

Strengths of the study include a universal healthcare setting providing equal access to services for all residents and using population-based high-quality registry data. This provides high coverage and data completeness reducing selection bias and increasing generalizability of the findings. To our knowledge, this is the only recent study investigating differences in CVD comparing curatively treated patients.

In conclusion, rates of AMI, cerebral infarction, and CVD death did not differ by treatment group the first years after treatment, indicating that ADT use in combination with radiotherapy may not increase the risk of these outcomes in a “real-world” curative setting. Further studies with individual data on ADT type and duration are needed to evaluate the effects of ADT duration on the risk of CVD. Increased overall CVD rate for definitive radiotherapy after the first year indicated a possible relationship between definitive radiotherapy and other CVDs, which should be further investigated.

## Data availability statement

The data analyzed in this study is subject to the following licenses/restrictions: Data in this study was accessed through linkage of data from official registers. Restrictions apply to the availability of these data, which were used under license for the current study, and they are as such not publicly available. Data are however available from the authors upon request and with permission from the Regional Committee for Medical Research and the respective registries. Requests to access these datasets should be directed to rachel.forster@fhi.no.

## Ethics statement

The studies involving human participants were reviewed and approved by The Regional Committee for Medical Research South-East approved the study (130363). Written informed consent for participation was not required for this study in accordance with the national legislation and the institutional requirements.

## Author contributions

RK obtained funding for the study. CK, RK, SH and TM arrived at the study concept and design. RK, RF and CK obtained the data. CK, RK and TM analyzed and interpreted the data. CK and RK drafted the manuscript. All authors contributed to the article and approved the submitted version.
