# Supplementary material for: Cardiovascular outcomes after curative prostate cancer treatment: A population-based cohort study
Source: Front Oncol. 2023 Mar 31;13:1121872. doi: 10.3389/fonc.2023.1121872 (PMC10102587; doi:10.3389/fonc.2023.1121872)
Supplement: Supplementary file 1 [file DataSheet_1.docx]

Supplementary Material

# Diagnosis codes used for outcomes according to the International classification of diseases version 10

- Overall CVD: I00-I99 or G45.
- Acute myocardial infarction (AMI): I21-I22.
- Cerebral infarction: I63.
- Thromboembolism: I80.2+I26.
- Previous CVD: I00-I99 or G45.
- Peripheral arterial disease: I70.2, I72.3, I72.4, I73.9

**Supplementary Figure 1.** Number and percentage of prostate cancer patients diagnosed 2010-2019 treated with radical prostatectomy or definitive radiotherapy**.**

**Supplementary Table 1. Cause-specific hazard ratios (CSHR) and Subdistribution hazard ratios (SHR) for CVD, AMI, cerebral infarction, thromboembolism and CVD specific mortality, comparing radiotherapy to prostatectomy**

| CVD | | | | | | | |
| --- | --- | --- | --- | --- | --- | --- | --- |
|  | Total period | | 1st year | | After 1st year | | Sensitivity 14 months^1^ |
|  | Unadjusted | Adjusted | Unadjusted | Adjusted | Unadjusted | Adjusted | Adjusted |
| TOTAL |  |  |  |  |  |  |  |
| CSHR (95% CI) |  |  | 1.37 (1.28-1.47) | 0.82 (0.76-0.89) | 1.72 (1.63-1.82) | 1.19 (1.11-1.28) | 1.30 (1.22-1.38) |
| SHR^1^ (95% CI) |  |  | 1.37 (1.28-1.46) | 0.83 (0.76-0.89) | 1.69 (1.60-1.79) | 1.18 (1.10-1.26) | 1.29 (1.21-1.37) |
| 1 month after^2^ CSHR (95% CI) | |  | 1.93 (1.78-2.08) | 1.17 (1.07-1.28) | 1.72 (1.63-1.82) | 1.21 (1.13-1.30) |  |
| LOW RISK |  |  |  |  |  |  |  |
| CSHR (95% CI) |  |  | 1.52 (1.04-2.22) | 0.93 (0.60-1.43) | 2.06 (1.60-2.65) | 1.37 (1.01-1.85) | 1.52 (1.16-2.00) |
| SHR (95% CI) |  |  | 1.52 (1.05-2.20) | 0.94 (0.61-1.44) | 2.04 (1.59-2.63) | 1.38 (1.01-1.89) | 1.53 (1.16-2.05) |
| INTERMEDIATE RISK | | | | | | | |
| CSHR (95% CI) |  |  | 1.49 (1.33-1.67) | 0.86 (0.76-0.98) | 1.80 (1.64-1.98) | 1.22 (1.09-1.37) | 1.34 (1.21-1.47) |
| SHR (95% CI) |  |  | 1.49 (1.34-1.67) | 0.87 (0.76-0.99) | 1.76 (1.60-1.94) | 1.20 (1.07-1.35) | 1.32 (1.19-1.46) |
| HIGH RISK LOCALIZED | | | | | | | |
| CSHR (95% CI) |  |  | 1.31 (1.16-1.48) | 0.79 (0.69-0.92) | 1.67 (1.51-1.85) | 1.18 (1.04-1.34) | 1.33 (1.19-1.48) |
| SHR (95% CI) |  |  | 1.31 (1.16-1.48) | 0.80 (0.69-0.93) | 1.65 (1.49-1.83) | 1.17 (1.03-1.33) | 1.32 (1.18-1.48) |
| HIGH RISK LOCALLY ADVANCED | | | | | | | |
| CSHR (95% CI) |  |  | 1.21 (1.06-1.37) | 0.80 (0.68-0.93) | 1.47 (1.32-1.64) | 1.13 (0.99-1.30) | 1.20 (1.06-1.35) |
| SHR (95% CI) |  |  | 1.21 (1.06-1.37) | 0.80 (0.68-0.93) | 1.45 (1.30-1.62) | 1.12 (0.97-1.28) | 1.18 (1.04-1.33) |
| AMI | | | | | | | |
|  | Total period | | 1st year | | After 1st year | Sensitivity 14 months^1^ | |
|  | Unadjusted | Adjusted | Unadjusted | Adjusted | Unadjusted | Adjusted | Adjusted |
| TOTAL | | | | | | | |
| CSHR (95% CI) | 1.59 (1.39-1.82) | 0.99 (0.83-1.19) |  |  |  |  | 1.04 (0.86-1.26) |
| SHR (95% CI) | 1.52 (1.33-1.74) | 0.98 (0.81-1.17) |  |  |  |  | Convergence not achieved |
| LOW RISK | | | | | | | |
| CSHR (95% CI) | 1.67 (0.92-3.03) | 1.19 (0.58-2.45) |  |  |  |  | Convergence not achieved |
| SHR (95% CI) | 1.65 (0.91-2.98) | 1.18 (0.55-2.55) |  |  |  |  | Convergence not achieved |
| INTERMEDIATE RISK | | | | | | | |
| CSHR (95% CI) | 1.68 (1.34-2.11) | 0.86 (0.64-1.15) |  |  |  |  | 0.92 (0.67-1.25) |
| SHR (95% CI) | 1.61 (1.29-2.03) | 0.84 (0.63-1.13) |  |  |  |  | 0.92 (0.68-1.23) |
| HIGH RISK LOCALIZED | | | | | | | |
| CSHR (95% CI) | 1.64 (1.28-2.10) | 1.10 (0.79-1.52) |  |  |  |  | 1.17 (0.82 -1.66) |
| SHR (95% CI) | 1.58 (1.23-2.03) | 1.09 (0.77-1.53) |  |  |  |  | 1.17 (0.83-1.65) |
| HIGH RISK LOCALLY ADVANCED | | | | | | | |
| CSHR (95% CI) | 1.31 (1.00-1.73) | 0.96 (0.68-1.35) |  |  |  |  | 0.98 (0.67-1.43) |
| SHR (95% CI) | 1.28 (0.97-1.67) | 0.94 (0.68-1.32) |  |  |  |  | 0.98 (0.68-1.42) |
| CEREBRAL INFARCTION | | | | | | | |
|  | Total period | | 1st year | | After 1st year | | Sensitivity 14 months^1^ |
|  | Unadjusted | Adjusted | Unadjusted | Adjusted | Unadjusted | Adjusted | Adjusted |
| TOTAL | | | | | | | |
| CSHR (95% CI) |  |  | 1.57 (1.05-2.33) | 0.77 (0.50-1.20) | 2.01 (1.70-2.38) | 1.11 (0.89-1.38) | 1.14 (0.92-1.42) |
| SHR (95% CI) |  |  | 1.57 (1.05-2.33) | 0.80 (0.52-1.22) | 1.92 (1.62-2.27) | 1.09 (0.87-1.36) | 1.12 (0.90-1.40) |
| LOW RISK | | | | | | | |
| CSHR (95% CI) | 2.29 (1.05-4.96) | 1.56 (0.60-4.10) |  |  |  |  | 1.56 (0.59-4.14) |
| SHR (95% CI) | 2.22 (1.02-4.81) | 1.55 (0.65-3.71) |  |  |  |  | 1.55 (0.63-3.78) |
| INTERMEDIATE RISK | | | | | | | |
| CSHR (95% CI) |  |  | 1.01 (0.49-2.01) | 0.40 (0.18-0.91) | 2.26 (1.70-3.01) | 1.15 (0.80-1.66) | 1.16 (0.81-1.67) |
| SHR (95% CI) |  |  | 1.00 (0.49-2.08) | 0.41 (0.18-0.90) | 2.16 (1.63-2.88) | 1.12 (0.78-1.60) | 1.13 (0.79-1.62) |
| HIGH RISK LOCALIZED | | | | | | | |
| CSHR (95% CI) | 1.52 (1.15-2.01) | 0.96 (0.66-1.39) |  |  |  |  | 1.05 (0.71-1.54) |
| SHR (95% CI) | 1.47 (1.11-1.94) | 0.95 (0.67-1.37) |  |  |  |  | 1.04 (0.70-1.54) |
| HIGH RISK LOCALLY ADVANCED | | | | | | | |
| CSHR (95% CI) | 1.85 (1.36-2.53) | 1.12 (0.75-1.66) |  |  |  |  | 1.08 (0.71-1.65) |
| SHR (95% CI) | 1.78 (1.31-2.43) | 1.09 (0.72-1.65) |  |  |  |  | 1.05 (0.68-1.63) |
| THROMBOEMBOLISM | | | | | | | |
|  | Total period | | 1st year | | After 1st year | | Sensitivity 14 months^1^ |
|  | Unadjusted | Adjusted | Unadjusted | Adjusted | Unadjusted | Adjusted | Adjusted |
| TOTAL | | | | | | | |
| CSHR (95% CI) |  |  | 0.43 (0.30-0.62) | 0.30 (0.20-0.44) | 1.62 (1.33-1.98) | 1.18 (0.92-1.52) | 1.11 (0.85-1.42) |
| SHR (95% CI) |  |  | 0.43 (0.30-0.62) | 0.30 (0.20-0.45) | 1.55 (1.27-1.90) | 1.15 (0.89-1.49) | 1.10 (0.85-1.42) |
| LOW RISK | | | | | | | |
| CSHR (95% CI) | 2.44 (1.12-5.32) | 1.87 (0.67-5.17) |  |  |  |  | 2.06 (0.73-5.86) |
| SHR (95% CI) | 2.37 (1.09-5.15) | 1.85 (0.64-5.32) |  |  |  |  | 2.04 (0.67-6.16) |
| INTERMEDIATE RISK | | | | | | | |
| CSHR (95% CI) |  |  | 0.56 (0.29-1.12) | 0.30 (0.13-0.68) | 1.41 (0.98-2.03) | 1.06 (0.67-1.68) | 1.04 (0.66-1.64) |
| SHR (95% CI) |  |  | 0.56 (0.29-1.11) | 0.30 (0.13-0.69) | 1.35 (0.94-1.95) | 1.03 (0.64-1.65) | 1.01 (0.64-1.60) |
| HIGH RISK LOCALIZED | | | | | | | |
| CSHR (95% CI) |  |  | 0.24 (0.12-0.51) | 0.20 (0.09-0.44) | 1.61 (1.13-2.29) | 1.17 (0.76-1.81) | 1.01 (0.66-1.56) |
| SHR (95% CI) |  |  | 0.24 (0.12-0.51) | 0.20 (0.09-0.43) | 1.55 (1.09-2.21) | 1.15 (0.74-1.80) | 1.00 (0.64-1.57) |
| HIGH RISK LOCALLY ADVANCED | | | | | | | |
| CSHR (95% CI) |  |  | 0.40 (0.23-0.69) | 0.42 (0.22-0.78) | 1.26 (0.86-1.83) | 1.18 (0.73-1.90) | 1.16 (0.74-1.82) |
| SHR (95% CI) |  |  | 0.40 (0.23-0.68) | 0.42 (0.22-0.81) | 1.22 (0.83-1.79) | 1.15 (0.70-1.89) | 1.14 (0.71-1.81) |
| CVD SPECIFIC MORTALITY | | | | | | | |
|  | Total period | | 1st year | | After 1st year | | Sensitivity 14 months^1^ |
|  | Unadjusted | Adjusted | Unadjusted | Adjusted | Unadjusted | Adjusted | Adjusted |
| TOTAL | | | | | | | |
| CSHR (95% CI) | 2.78 (2.15-3.61) | 1.12 (0.79-1.59) |  |  |  |  | 1.11 (0.77-1.60) |
| SHR (95% CI) | 2.68 (2.07-3.47) | 1.10 (0.77-1.58) |  |  |  |  | 1.09 (0.74-1.61)) |
| INTERMEDIATE RISK | | | | | | | |
| CSHR (95% CI) | 2.48(1.61-3.82) | 1.19 (0.68-2.10) |  |  |  |  | 1.32 (0.73-2.40) |
| SHR (95% CI) | 2.39 (1.56-3.68) | 1.17 (0.66-2.08) |  |  |  |  | 1.30 (0.70-2.42) |
| HIGH RISK LOCALIZED | | | | | | | |
| CSHR (95% CI) | 2.50 (2.60-3.92) | 0.92 (0.50-1.69) |  |  |  |  | 0.93 (0.49-1.76) |
| SHR (95% CI) | 2.42 (1.54-3.79) | 0.90 (0.49-1.64) |  |  |  |  | 0.91 (0.48-1.72) |
| HIGH RISK LOCALLY ADVANCED | | | | | | | |
| CSHR (95% CI) | 3.46 (1.89-6.32) | 1.17 (0.57-2.41) |  |  |  |  | 1.03 (0.47-2.26) |
| SHR (95% CI) | 3.36 (1.84-6.13) | 1.13 (0.52-2.48) |  |  |  |  | 1.01 (0.43-2.39) |

Adjusted for age, previous cardiovascular disease, cT stage, cN stage, Gleason score, PSA, Charlson comorbidity index, WHO performance status, healthcare region, diagnosis year. ^1^Sensitivity analysis starting follow-up from 14 months after diagnosis for all patients. ^2^Follow-up starting 1 month after treatment.

**Supplementary Table 2. Cause-specific hazard ratios of CVD, AMI, cerebral infarction, thromboembolism and CVD specific mortality, comparing radiotherapy to prostatectomy and covariates age, previous CVD, Charlson comorbidity index (CCI) and WHO performance status.**

| **CVD** | | | | | | |
| --- | --- | --- | --- | --- | --- | --- |
|  | | Total  aCSHR (95% CI) | Low risk  aCSHR (95% CI) | Intermediate risk  aCSHR (95% CI) | High risk localized  aCSHR (95% CI) | High risk  locally advanced  aCSHR (95% CI) |
| Treatment (ref: prostatectomy) | |  |  |  |  |  |
|  | 1st year | 0.82 (0.76-0.89) | 0.93 (0.60-1.43) | 0.86 (0.76-0.98) | 0.79 (0.69-0.92) | 0.80 (0.68-0.93) |
|  | After 1st year | 1.19 (1.11-1.28) | 1.37 (1.01-1.85) | 1.22 (1.09-1.37) | 1.18 (1.04-1.34) | 1.13 (0.99-1.30) |
| Age (5-year interval) | | 1.17 (1.14-1.19) | 1.14 (1.06-1.23) | 1.18 (1.14-1.22) | 1.15 (1.11-1.20) | 1.17 (1.12-1.21) |
| Previous CVD (ref: no) | |  |  |  |  |  |
|  | 1st year | 3.14 (2.90-3.39) | 2.56 (1.99-3.28)^1^ | 3.23 (2.85-3.66) | 3.07 (2.67-3.54) | 3.03 (2.62-3.51) |
|  | After 1st year | 1.19 (1.11-1.28) |  | 1.92 (1.70-2.16) | 1.99 (1.73-2.29) | 2.06 (1.79-2.37) |
| CCI (ref: 0) | |  |  |  |  |  |
|  | 1 | 1.17 (1.09-1.25) | 1.06 (0.77-1.45) | 1.29 (1.16-1.44) | 1.16 (1.03-1.31) | 1.05 (0.92-1.20) |
|  | 2 | 1.44 (1.31-1.58) | 1.22 (0.77-1.92) | 1.44 (1.24-1.69) | 1.62 (1.36-1.93) | 1.28 (1.07-1.53) |
|  | ≥3 | 1.53 (1.33-1.76) | 1.22 (0.58-2.54) | 1.89 (1.51-2.37) | 1.56 (1.18-2.06) | 1.24 (1.04-1.28) |
| WHO perf. status (ref: 0) | |  |  |  |  |  |
|  | 1 | 1.25 (1.17-1.33) | 1.16 (0.82-1.64) | 1.32 (1.18-1.48) | 1.23 (1.10-1.39) | 1.21 (1.08-1.35) |
|  | ≥2 | 1.17 (1.02-1.33) | 1.15 (0.58-2.27) | 1.12 (0.90-1.40) | 1.29 (1.01-1.66) | 1.12 (0.87-1.45) |
| **AMI** | | | | | | |
|  | | Total  aCSHR (95% CI) | Low risk  aCSHR (95% CI) | Intermediate risk  aCSHR (95% CI) | High risk localized  aCSHR (95% CI) | High risk  locally advanced  aCSHR (95% CI) |
| Treatment (ref: prostatectomy) | | 0.99 (0.83-1.19) | 1.19 (0.58-2.45) | 0.86 (0.64-1.15) | 1.10 (0.79-1.52) | 0.96 (0.68-1.35) |
| Age (5-year interval) | | 1.21 (0.83-1.19) | 1.19 (0.58-2.45) | 1.37 (1.22-1.53) | 1.22 (1.08-1.38) | 0.96 (0.68-1.35) |
| Previous CVD (ref: no) | |  |  |  |  |  |
|  | 1st year | 1.35 (1.13-1.61)^1^ | 1.37 (0.68-2.77)^1^ | 1.86 (1.04-3.33) | 0.98 (0.68-1.41)^1^ | 1.78 (1.27-2.48)^1^ |
|  | After 1st year |  |  | 1.23 (0.89-1.69) |  |  |
| CCI (ref: 0) | |  |  |  |  |  |
|  | 1 | 1.26 (1.02-1.55) | 2.62 (1.26-5.43) | 1.36 (0.96-1.93) | 1.02 (0.67-1.56) | 1.20 (0.80-1.80) |
|  | 2 | 1.98 (1.51-2.59) | 1.91 (0.52-6.99) | 2.21 (1.43-3.40) | 1.90 (1.14-3.18) | 1.84 (1.11-3.05) |
|  | ≥3 | 1.78 (1.16-2.73) | - | 2.81 (1.53-5.52) | 1.69 (0.72-3.96) | 1.08 (0.45-2.63) |
| WHO perf. status (ref: 0) | |  |  |  |  |  |
|  | 1 | 1.29 (1.26-1.58) | 1.74 (0.72-4.16) | 1.66 (1.20-2.30) | 1.04 (0.72-1.50) | 1.12 (0.77-1.62) |
|  | ≥2 | 1.63 (1.15-2.73) | 0.86 (0.04-2.52) | 2.28 (1.38-3.75) | 1.09 (0.72-3.96) | 1.45 (0.73-2.90) |
| **CEREBRAL INFARCTION** | | | | | | |
|  | | Total  aCSHR (95% CI) | Low risk  aCSHR (95% CI) | Intermediate risk  aCSHR (95% CI) | High risk localized  aCSHR (95% CI) | High risk  locally advanced  aCSHR (95% CI) |
| Treatment (ref: prostatectomy) | |  |  |  |  |  |
|  | 1st year | 0.77 (0.50-1.20) | 1.56 (0.60-4.10)^3^ | 0.40 (0.18-0.91) | 0.96 (0.66-1.39)^3^ | 1.12 (0.75-1.66)^3^ |
|  | After 1st year | 1.11 (0.89-1.38) |  | 1.15 (0.80-1.66) |  |  |
| Age (5-year interval) | | 1.44 (1.32-1.56) | 1.84 (1.25-2.70) | 1.54 (1.33-1.77) | 1.29 (0.66-1.39) | 1.12 (0.75-1.66) |
| Previous CVD (ref: no) | | 1.46 (1.19-1.79) | 1.28 (0.39-4.20) | 1.42 (1.01-1.99) | 1.83 (1.27-2.63) | 1.24 (0.85-1.81) |
| CCI (ref: 0) | |  |  |  |  |  |
|  | 1 | 1.17 (1.19-1.79) | 0.62 (0.13-2.98) | 1.44 (0.97-2.14) | 0.86 (0.54-1.35) | 1.27 (0.82-1.96) |
|  | 2 | 1.23 (0.87-1.74) | 1.05 (0.12-8.93) | 1.46 (0.82-2.61) | 1.18 (0.63-2.20) | 1.01 (0.53-1.89) |
|  | ≥3 | 1.57 (0.98-2.54) | 5.74 (0.87-38.0) | 2.16 (0.99-4.71) | 0.91 (0.31-2.69) | 1.30 (0.58-2.93) |
| WHO perf. status (ref: 0) | |  |  |  |  |  |
|  | 1 | 1.28 (1.02-1.60) | 0.39 (0.46-3.32) | 1.24 (0.83-1.87) | 1.54 (1.05-2.26) | 1.21 (0.82-1.80) |
|  | ≥2 | 1.58 (1.06-2.37) | - | 1.90 (1.01-3.57) | 1.42 (0.62-3.28) | 1.89 (0.94-3.79) |
| **THROMBOEMBOLISM** | | | | | | |
|  | | Total  aCSHR (95% CI) | Low risk  aCSHR (95% CI) | Intermediate risk  aCSHR (95% CI) | High risk localized  aCSHR (95% CI) | High risk  locally advanced  aCSHR (95% CI) |
| Treatment (ref: prostatectomy) | |  |  |  |  |  |
|  | 1st year | 0.30 (0.20-0.44) | 1.87 (0.67-5.17)^1^ | 0.30 (0.13-0.68) | 0.20 (0.09-0.44) | 0.42 (0.22-0.78) |
|  | After 1st year | 1.18 (0.92-1.52) |  | 1.06 (0.67-1.68) | 1.17 (0.76-1.81) | 1.18 (0.73-1.90) |
| Age (5-year interval) | | 1.13 (1.04-1.22) | 1.15 (0.82-1.64) | 1.13 (0.98-1.30) | 1.14 (0.99-1.31) | 1.08 (0.94-1.25) |
| Previous CVD (ref: no) | |  |  |  |  |  |
|  | 1st year | 1.78 (1.24-2.55) | 0.69 (0.18-2.69)^1^ | 2.57 (1.43-4.63) | 1.19 (0.79-1.79)^1^ | 1.22 (0.80-1.85)^1^ |
|  | After 1st year | 0.96 (0.72-1.28) |  | 0.74 (0.43-1.28) |  |  |
| CCI (ref: 0) | |  |  |  |  |  |
|  | 1 | 1.03 (0.75-1.63) | 1.26 (0.32-4.97) | 0.74 (0.43-1.29) | 0.97 (0.60-1.56) | 1.04 (0.64-1.71) |
|  | 2 | 1.10 (0.72-1.70) | - | 1.46 (0.79-2.72) | 1.22 (0.63-2.38) | 0.86 (0.41-1.82) |
|  | ≥3 | 0.10 (0.01-0.71) | - | 0.31 (0.04-2.31) | 0.28 (0.04-2.13) |  |
| WHO perf. status (ref: 0) | |  |  |  |  |  |
|  | 1 | 1.23 (0.92-1.63) | 2.22 (0.64-7.72) | 1.57 (0.99-2.48) | 1.07 (0.68-1.68) | 1.02 (0.64-1.62) |
|  | ≥2 | 0.68 (0.32-1.45) | - | 0.53 (0.13-2.17) | 1.32 (0.53-3.28) | 0.24 (0.03-1.76) |
| **CVD SPECIFIC MORTALITY** | | | | | | |
|  | | Total  aCSHR (95% CI) | Low risk  aCSHR (95% CI) | Intermediate risk  aCSHR (95% CI) | High risk localized  aCSHR (95% CI) | High risk  locally advanced  aCSHR (95% CI) |
| Treatment (ref: prostatectomy) | | 1.12 (0.79-1.59) | - | 1.19 (0.68-2.10) | 0.92 (0.50-1.69) | 1.17 (0.57-2.41) |
| Age (5-year interval) | | 1.45 (1.27-1.66) |  | 1.38 (1.09-1.74) | 1.61 (1.26-2.05) | 1.35 (1.06-1.73) |
| Previous CVD (ref: no) | | 1.56 (1.13-2.14) |  | 1.48 (0.86-2.55) | 1.78 (0.99-3.17) | 1.53 (0.85-2.76) |
| CCI (ref: 0) | |  |  |  |  |  |
|  | 1 | 2.06 (1.45-2.93) |  | 2.50 (1.41-4.46) | 1.42 (0.74-2.71) | 2.60 (1.35-5.00) |
|  | 2 | 2.05 (1.25-3.39) |  | 1.16 (0.39-3.45) | 1.69 (0.66-4.31) | 3.78 (1.72-8.32) |
|  | ≥3 | 2.97 (1.55-5.69) |  | 3.32 (1.18-9.36) | 5.67 (1.97-16.29) | 1.46 (0.31-6.82) |
| WHO perf. status (ref: 0) | |  |  |  |  |  |
|  | 1 | 1.88 (1.37-2.59) |  | 2.22 (1.29-3.83) | 1.81 (1.03-3.16) | 1.68 (0.93-3.04) |
|  | ≥2 | 2.22 (1.55-3.79) |  | 0.70 (0.17-2.96) | 3.82 (1.64-8.88) | 2.88 (1.21-6.82) |

Adjusted for age, previous cardiovascular disease, cT stage, cN stage, Gleason score, PSA, Charlson comorbidity index, WHO performance status, healthcare region, diagnosis year. ^1^Whole period

Risk groups defined according to the European Association of Urology (EAU) Guidelines: 1) low risk PCa: PSA <10 ng/ml and Gleason score <7 and cT1-2a; 2) intermediate risk PCa: PSA 10-20 ng/ml or Gleason score 7 or cT2b; 3) high risk localized PCa: PSA >20 ng/ml or Gleason score >7 or cT2c; 4) high risk locally advanced PCa: cT3-4 or N1 with any PSA and Gleason score.

**Supplementary Table 3. Cause-specific hazard ratios (aCSHR) of CVD after treatment with definitive radiotherapy compared to radical prostatectomy (ref).**

| **CVD** | | | |
| --- | --- | --- | --- |
|  | | Total | High risk *PCa* |
|  | | aCSHR (95% CI | aCSHR (95% CI) |
| *No previous CVD* | | 1.04 (0.97-1.11)* | 0.97 (0.89-1.06)* |
|  | *1^st^ year* | 0.75 (0.68-0.84) | 0.69 (0.60-0.79) |
|  | *After 1^st^ year* | 1.22 (1.12-1.32) | 1.17 (1.06-1.30) |
| *Previous CVD* | | 1.00 (0.91-1.10) | 1.02 (0.89-1.16) |
| **AMI** | | | |
|  | | Total | High risk *PCa* |
|  | | aCSHR (95% CI) | aCSHR (95% CI) |
| *No previous CVD* | | 0.92 (0.74-1.14) | 1.02 (0.77-1.34) |
| *Previous CVD* | | 1.19 (0.87-1.63) | 1.13 (0.72-1.78) |
| **THROMBOEMBOLISM** | | | |
|  | | Total | High risk *PCa* |
|  | | aCSHR (95% CI) | aCSHR (95% CI) |
| *No previous CVD* | | 0.85 (0.69-1.06)* | 0.78 (0.60-1.02)* |
|  | *1^st^ year* | 0.30 (0.20-0.45) | 0.27 (0.17-0.44) |
|  | *After 1^st^ year* | 1.26 (0.99-1.59) | 1.27 (0.93-1.72) |
| *Previous CVD* | | 0.67 (0.47-0.97) | 0.70 (0.44-1.12) |
| **CEREBRAL INFARCTION** | | | |
|  | | Total | High risk *PCa* |
|  | | aCSHR (95% CI | aCSHR (95% CI) |
| *No previous CVD* | | 1.00 (0.78-1.28) | 0.93 (0.68-1.28) |
| *Previous CVD* | | 1.16 (0.80-1.69) | 1.34 (0.79-2.26) |
| **CVD MORTALITY** | | | |
|  | | Total | High risk *PCa* |
|  | | aCSHR (95% CI | aCSHR (95% CI) |
| *No previous CVD* | | 0.98 (0.63-1.52) | 1.10 (0.61-1.98) |
| *Previous CVD* | | 1.41 (0.79-2.51) | 0.97 (0.47-1.98) |

Adjusted for age, cT stage, cN stage, Gleason score, PSA, Charlson comorbidity index, WHO performance status, healthcare region, diagnosis year.

*PH assumption invalid
